# *Alnus nitida* and urea-doped *Alnus nitida*-based silver nanoparticles synthesis, characterization, their effects on the biomass and elicitation of secondary metabolites in wheat seeds under *in vitro* conditions

**DOI:** 10.1016/j.heliyon.2023.e14579

**Published:** 2023-03-14

**Authors:** Sajad Khan, Raham Sher Khan, Muhammad Zahoor, Sikandar khan, Noor Ul Islam, Tariq Khan, Zar Muhammad, Riaz Ullah, Ahmed Bari

**Affiliations:** a Department of Biotechnology, Abdul Wali Khan University Mardan, Mardan 23200, Pakistan; b Department of Chemistry, Faculty of Chemical Engineering, Istanbul University Avcilar Campus, Istanbul, Turkey; c Department of Biochemistry, University of Malakand, Chakdara 18800, Pakistan; d Department of Chemistry, University of Malakand, Chakdara 18800, Pakistan; e School of Nanoscience and Nano-engineering University of North Carolina, USA; f Department of Biotechnology, University of Malakand, Chakdara 18800, Pakistan; g Quality Enhancement Cell, University of Malakand, Chakdara 18800, Pakistan; h Department of Pharmacognosy, College of Pharmacy, King Saud University, Riyadh, Saudi Arabia; i Department of Pharmaceutical Chemistry, College of Pharmacy, King Saud University, Riyadh, Saudi Arabia

**Keywords:** Silver nanoparticles, Urea-doped silver nanoparticles, Wheat, Urea, Secondary metabolites

## Abstract

Nano-fertilizers are superior to conventional fertilizers, but their effectiveness has not yet been adequately explored in the field of agriculture. In this study, silver nanoparticles using leaves extract of an *Alnus nitida* plant were synthesized and further doped with urea to enhance the plant biomass and metabolic contents. The synthesized *Alnus nitida* silver nanoparticles (A.N-AgNPs) and urea-doped silver nanoparticles (U-AgNPs) were characterized using Scanning Electron Microscopy, Transmission Electron Microscopy, Powder X-ray Diffraction, and Energy Dispersive X-ray. The wheat seeds were grown in media under controlled conditions in the plant growth chamber. The effectiveness of nanoparticles was studied using different A.N-AgNPs and U-AgNPs concentrations (0.75 μg/ml, 1.5 μg/ml, 3 μg/ml, 6 μg/ml, and 15 μg/ml). They were compared with a control group that received no dose of nanoparticles. The plant biomass, yield parameters, and wheat quality were analyzed. The effect of silver nanoparticles and U-AgNPs were examined in developing wheat seeds and their potency in combating biotic stresses such as nematodes, herbivores, fungi, insects, weeds and bacteria; abiotic stresses such as salinity, ultraviolet radiation, heavy metals, temperature, drought, floods etc. In the seedlings, six possible phytochemicals at a spray dose of 6 μg/ml of U-AgNPs were identified such as dihydroxybenzoic acids, vanillic acid, apigenin glucosidase, *p*-coumaric acid, sinapic acid, and ferulic acid whereas in other treatments the number of phenolic compounds was lesser in number as well as in concentrations. Moreover, various parameters of the wheat plants, including their dry weight and fresh weight, were assessed and compared with control group. The findings of the study indicated that A.N-AgNPs and U-AgNPs act as metabolite elicitors that induced secondary metabolite production (total phenolic, flavonoid, and chlorophyll contents). In addition, U-AgNPs provided a nitrogen source and were considered a smart nitrogen fertilizer that enhanced the plant biomass, yields, and metabolite production.

## 1 Introduction

The surging demand for food driven by a growing global population with elevated living standards poses considerable challenges for agriculture and has a profound impact on the environment. The world is experiencing an alarmingly high growth rate in its population. According to the latest statistics, the world's population has surpassed 7.884 billion peoples. The rapid growth rate become a serious concern for humanity to provide food to the massive population [1]. In the next few decades, global food demand will surge to 62% and the hunger risk to 30%, which would be an alarming situation for the world [2]. The extensive application of chemical fertilizers has been a key contributor to improving crop productivity. Nevertheless, this practice has given rise to significant environmental pollution concerns [3]. Proper fertilizer management in the field is one of the most significant challenges, mainly focusing on the maximum nutritional efficacy of fertilizers to induce crop yield and ensure environmental safety [4]. Urea plays a vital role as a nitrogen fertilizer and serves as a critical component of chemical fertilizers that are indispensable for agricultural practices [5]. It is interesting to know that nitrogen fertilizer utilization had increased to 800% since 1961 [6].

Elicitation is the most efficacious technique *in vitro* culture for inducing the production of secondary metabolites in various medicinal plants [7]. The nanoparticles act as abiotic elicitors to enhance the production of secondary metabolites by performing oxidative stress-relieving effects in the plant cells, improving antioxidant potential and metabolism [8,9]. The rapid expansion of the world's population and the recent global outbreak of COVID-19 are aggravating the problem of food scarcity, making it an increasingly pressing issue. These challenges are compelling researchers to develop more effective and sustainable techniques for crop production, which can cater to the growing food demands of humanity [10]. Thus, scientists have tried various approaches in which nanotechnology was proven highly effective with less toxicity. The environmental concerns associated with conventional fertilizers include water pollution, soil degradation, greenhouse gas emissions, and negative impacts on biodiversity. Developing a novel form of nanotechnologies-based nano-fertilizers is one of the promising approaches to significantly enhance wheat plant yield and quality, reducing the environmental issues (negative impacts on soil fertility, water, and air quality; causes greenhouse gas emissions, eutrophication, and health problem) associated with conventional fertilization. Smart fertilizers improve plant biomass, yields, and response against biotic and abiotic stresses [[11], [12], [13]]. In this way, fertilizers doped with nanoparticles act as nano fertilizers, as they not only supply the required amount of nitrogen to plants, but also protect them from microbial infections and environmental stresses [14,15].

The selection of *Alnus nitida* leaves extract for the biosynthesis of silver nanoparticles was based on various factors such as easily accessible, a rich source of plant secondary metabolites, such as flavonoids, alkaloids, tannins, and various biomolecules, such as proteins, enzymes, and sugars, reducing silver ions and synthesizing silver nanoparticles with a high degree of uniformity and stability [16]. The phytochemical compounds of *Alnus nitida* transfer their electrons to the metal ion resulting in the synthesis of nanoparticles, and quercetin has been identified as the compound responsible for the reduction process [17]. There are various physical, chemical, and biological techniques available for the synthesis of inorganic nanoparticles, including chemical reduction, sol-gel synthesis, hydrothermal synthesis, and laser ablation. However, green synthesis is a novel technique to produce nanoparticles, which employs natural, non-toxic, and sustainable materials like plant extracts, bacteria, or fungi. This approach provides significant benefits over conventional methods, which use hazardous chemicals and high-energy processes [18]. The central hypothesis driving green synthesis is that by utilizing natural, non-toxic, and sustainable materials, it is possible to develop a safer, more environmentally friendly, and cost-effective approach for producing nanoparticles that possess the desired properties for various applications [19]. It involves the identification and optimization of green synthesis methods to improve the efficiency, stability, and scalability of the process. Moreover, it allows for better control over the shape, size, and properties of the nanoparticles produced during the reaction. Therefore, biological synthesis holds significant promise as a superior alternative to traditional physical and chemical approaches to synthesized inorganic nanoparticles [20]. Silver nanoparticles are valuable in various fields, such as biomedicine, environmental remediation, and consumer products, due to their distinct physical and chemical properties. Their high surface area-to-volume ratio enables them to be highly reactive and interact with a broad range of molecules, making them a versatile material [21,22].

The present attempt made herein is novel as for the first time silver nanoparticles and urea-doped silver nanoparticles were synthesized using *Alnus nitida*-based leaves extract. The newly synthesized urea-doped silver nanoparticles (U-AgNPs) and silver nanoparticles (A.N-AgNPs) were subjected to wheat seeds under *in vitro* conditions. Moreover, we conducted a thorough examination of the biochemical and physiological effects of A.N-AgNPs and U-AgNPs in wheat seeds. The utilization of this innovative approach is a significant step forward in the field of silver nanoparticle applications. By utilizing plant extract and doping the nanoparticles with urea, the efficacy of the nanoparticles is significantly enhanced. The results of the study have the potential to revolutionize the field of nanoparticle synthesis and its applications in agriculture.

## 2 Material and methods

### 2.1 Preparation of leaf in powdered form

To prepare the powder from *Alnus nitida*, fresh leaves were collected and washed meticulously with distilled water to eliminate any impurities. Afterward, they were dried in a well-ventilated area or a low-temperature oven (below 60 °C) to prevent the loss of bioactive compounds. Once dry, the leaves were finely ground into a powder using a mortar and pestle. The resulting powder was then transferred into a clean, dry container and stored in a cool, dark location until needed to synthesize silver nanoparticles.

### 2.2 Preparation of *Alnus nitida* extract

The fresh leaves of *Alnus nitida* plant were collected from the upper hilly area of the Ziarat Talash region of District Dir lower, Khyber Pakhtunkhwa Pakistan. The plant was identified by a taxonomist in the Herbarium; Department of Botany Abdul Wali Khan University Mardan, Pakistan. To prepare the extract of *Alnus nitida* leaves, 10 g of fresh leaves were weighed and added to a 500 ml beaker containing 100 ml of distilled water. The beaker was then heated to 60 °C and maintained at this temperature for 10 min before being decanted. The solution was filtered using Whatman Filter Paper No. 1, and the resulting filtrate was centrifuged at 15,000 rpm for 20 min. The resulting pellet was then dispersed in distilled water and kept for further use.

### 2.3 Chemicals

Silver nitrate (AgNO 3), and all other solvents and chemicals used in the study were of the highest purity and analytical grade. These chemicals were provided by Sigma Aldrich.

### 2.4 Preparation of silver nitrate solution

The solution of 4 mM silver nitrate was prepared in deionized distilled water. Subsequently, a 0.4 mM silver nitrate solution was prepared through dilution from the aforementioned stock solution and stored in a jar for further synthesis of nanoparticles.

### 2.5 Biosynthesis of silver nanoparticles using *Alnus nitida* extract

The silver nitrate solution (0.4 mM) of 30 ml was added to 10 ml of *Alnus nitida* leaves extract and the synthesis of silver nanoparticles was performed by adding 30 ml of 0.4 mM of silver nitrate solution to 10 ml of *Alnus nitida* leaves extract. The reaction was carried out under strict dark conditions to prevent the photoactivation of silver nitrate. After 12 h, the solution changed its color, confirming the formation of silver nanoparticles. The solution containing silver nanoparticles was subjected to centrifugation at 4400 rpm and 25 °C for 15 min. The residues were recovered from the solution and dried at room temperature. The fine powder was obtained and stored for further analysis [[23], [24], [25]].

### 2.6 Urea-doped silver nanoparticles

The synthesis of urea-doped silver nanoparticles was performed using the protocol of Carmona et al. [26]*.* The silver nanoparticles solution was heated to 37 °C for 5 min*.* The solution suspension was centrifuged at 4500 rpm for 10 min and washed (300 ml × 2). Urea was poured into the water (1.0 g in 6 ml) and mixed vigorously with slurry (obtained silver nanoparticles) to obtain a homogenate mixture. After homogenization, the sample was frozen and lyophilized. The powder sample was obtained which was stored at 4 °C for further studies [27].

### 2.7 Plant material and culture conditions

To sterilize the wheat seeds, they were subjected to 0.1% mercuric chloride solution and were kept on filter paper (3–5 min) till dryness to enhance germination speed [28]. Murashige and Skoog media (MS Media, M519 Phytotech lab, USA) were used for seeds growth [29]. Therefore, a total of 1.4 g of MS media was carefully measured and combined with 10.5 g of sucrose and 2.8 g of agar in 350 ml of distilled water. The pH of the media was adjusted to 5.2–5.8. The 350 ml of the synthesized growth medium was distributed evenly into seven separate flasks, and each flask was covered with both cotton and aluminum foil. The flasks were then autoclaved to eliminate any potential contaminants that could negatively affect the growth of wheat plants. After the autoclaving process, the flasks were left undisturbed overnight to allow the growth medium to cool to room temperature and attain optimal conditions for subsequent analysis.

The next step involved transferring the sterilized wheat seeds to each of the prepared growth medium-filled flasks, which were then securely plugged with cotton to ensure sterility. The flasks were then thoroughly covered with aluminum foil to prevent any potential contamination of the seeds. These seed-supplemented flasks were subsequently transferred to a growth chamber (BioBase, BJPX-A1500C, Ltd. China) for germination. The growth chamber was maintained at a temperature of 25 ± 2 °C and a humidity of 70%, while the illumination was set to 16 h of light followed by 8 h of darkness. The light was provided by Philips TLD 35 fluorescent lamps, with an intensity of 40 μmol m^−2^ s^−1^ [30].

### 2.8 Dispersion and supplementation of media with A.N-AgNPs and urea-doped silver nanoparticles

The dispersion of the synthesized A.N-AgNPs and U-AgNPs was prepared at varying concentrations for the MS media at 0.75 μg/ml, 1.5 μg/ml, 3 μg/ml, 6 μg/ml, and 15 μg/ml per 350 ml and the control with no nanoparticles. A.N-AgNPs and U-AgNPs were dispersed properly in solution using sonication for 1 h. Afterward, the diverse range of A.N-AgNPs and U-AgNPs were supplemented to the cultured wheat seeds on MS media. In this experiment, the flask without A.N-AgNPs and U-AgNPs were designated as the control. The flasks were transferred to a growth chamber to allow the growth of the seeds. Samples from the seed cultures were harvested on the 10th, 20th, 30th, and 40th days after the initial transfer of the seeds. The fresh weight of the wheat plant was accurately measured using an analytical balance (AW 120, Electronic balance) after each harvest. The plant was then dried overnight at 35 °C in an oven, and the dry weight was recorded. Subsequently, the wheat plant was finely ground to a powder and stored for further analysis. The wheat seeds were collected and thoroughly washed to remove any impurities.

## 3 Analysis of the secondary metabolites

### 3.1 Total phenolic content assay

To prepare the samples for analysis, a multi-channel micropipette was used to load 20 μL of the sample into each of the 96 wells of a microplate. This was followed by the addition of 90 μL of Folin-Ciocalteu reagent to each well, and then 90 μL of sodium carbonate was added to complete the reaction mixture. The microplate was incubated for 30 min. The positive control in this process was different concentrations of gallic acid (25, 20, 15, 10, and 5 μg/ml). A microplate reader was used to record samples absorbance and determine total phenolic content. Each step of the experiment was performed in triplicate.

### 3.2 Total flavonoids content assay

To determine the total flavonoid content, 20 μL of the sample was added to a 96-well microplate, along with 10 μL of aluminum chloride, 10 μL of potassium acetate, and 160 μL of distilled water. The microplate was incubated for 30 min. The positive control in this process was the final concentrations of Quercetin (40, 20, 10, 5, and 2.5 μg/ml). A microplate reader was used to record the absorbance of samples. Every step in this experiment was performed in triplicate.

### 3.3 Pigments contents assay

The spectrophotometer at 646.8, 663.2, and 470 nm was used to measure the values of chlorophyll *a* (Ca), chlorophyll *b* (Cb), and carotenoids (Cx + c) [31]. To prepare the wheat plant extract, a wheat plant was carefully collected and ground to produce 1.0 ml of juice. The juice was then mixed with 4.0 ml of acetone and 50 mg of CaCO_3_, after which the mixture was allowed to stand for 5 min. To remove any insoluble particles, the mixture was then centrifuged at 10,000 rpm for 5 min. The absorbances of the resulting supernatant were measured at 646.8 nm, 663.2 nm, and 470 nm, respectively. The quantities of pigments contained in the juice samples were calculated using equations (1), (2), (3), given below:

(1) $$C_{a}=12.25A_{663.2}-2.79A_{646.8}$$

(2) $$C_{b}=21.50A_{646.8}-5.10A_{663.2}$$

(3) $$C_{x+c}=1000A_{470}-182C_{a}-85.02C_{b}/198$$

### 3.4 Determination of antioxidative potential of wheat plants extract in response to A.N-AgNPs and U-AgNPs

The Diphenyl Picryl Hydrazyl (DPPH) assay was used to determine antioxidant potential (free radicle scavenging activity) of wheat plant extract that received diverse treatments of silver and urea-doped silver nanoparticles. About 20 μL samples and a DPPH reagent of 180 μL were loaded into microplate wells. Afterward, samples were incubated for 1 h. Ascorbic acid was used as a positive control with different concentrations (0.75, 1.5, 3, 6, and 15 μg/ml). A microplate reader was used to record the absorbance of samples. Every step of the experiment was performed in triplicate [10,25].

### 3.5 HPLC-UV characterization

High-performance liquid chromatography with ultraviolet detection (HPLC-UV) was utilized for the characterization and quantification of the sample using established analytical techniques [32]. The wheat plant was crushed with a mortar and pestle, and then approximately 1 g of powder was equally mixed with methanol. The resulting mixture was then subjected to a water bath at a temperature of 70 °C for 1 h. The mixture was centrifuged at 4000 rpm for 10 min and then filtered with Whatman filter paper. HPLC-UV determines the presence of total phenolic and flavonoid content in the plant samples. The wavelength was set to 320 nm for total phenolic content, and the chromatograms were arranged from 190 to 500 nm.

### 3.6 Statiscial analyses

In our study, we implemented a completely randomized design to perform experiments. These experiments were repeated twice with three replicates in each experiment to ensure statistical accuracy. We employed a linear regression analysis to determine significant mean differences (P \2 0.05). We utilized the latest version of SPSS (version 20) for carrying out the statistical analysis, which helped us to obtain precise and accurate results. All of the figures in this study was prepared by origin 9.0.

## 4 Characterization of silver nanoparticles and urea-doped silver nanoparticles

### 4.1 UV–vis spectroscopy

A UV–visible spectrophotometer (UV-2450, Shimadzu, Japan) was used to analyze the UV–Vis spectra of silver nanoparticles. A 1 nm resolution was used to examine the reduction of Ag ^+^ to Ag^0^ by leaves extract. The mixture of AgNO_3_ and leaves extract was incubated for 24 h. The spectra were arranged between 200 and 800 nm. The background accuracy of experiments was corrected using double distilled water.

### 4.2 Powder X-ray diffraction (PXRD)

The mixture of A.N-AgNPs and U-AgNPs was subjected to centrifugation at 10,000 rpm for 15 min. The resulting pellets were washed with sterile double distilled water and then centrifuged again at 10,000 rpm for 10 min. The purified pellets of A.N-AgNPs and U-AgNPs were dried in an oven at 50 °C and subjected to analysis using an X-ray Diffraction Unit (XRD) (Pan Analytical, X-pert pro, China). The powdered samples were analyzed using a Cu-Kα radiation source with a range of 20–80°, and the presence of phase variety, crystalline nature, and grain size of the samples was evaluated using X-ray diffraction spectroscopy.

### 4.3 Transmission Electron Microscopy (TEM)

TEM images were obtained using a TALOS transmission electron microscope with a voltage of 200 kV. Initially, the samples were dispersed in MiliQ water and then subjected to 15 min of sonication. The resulting samples were drop-cast onto carbon-coated copper grids (300 mesh) and incubated at room temperature for 1 h. Energy-dispersive X-ray analysis (EDX) was conducted on a TALOS machine using a high-energy beam at a voltage of 40 kV.

#### 4.3.1 Scanning Electron Microscopy (SEM)

The SEM images were obtained using a focused-ion beam scanning electron microscope that was equipped with SE detectors. The voltage used for our samples was approximately 20 kV. Before imaging, the powdered samples were spread onto a black carbon tape surface on steel grids and coated with an ultrathin layer of electrically conducting gold metal [33].

## 5 Results and discussion

### 5.1 Characterization of the silver nanoparticles

#### 5.1.1 Formation of dark brown color

**Fig. 1:**
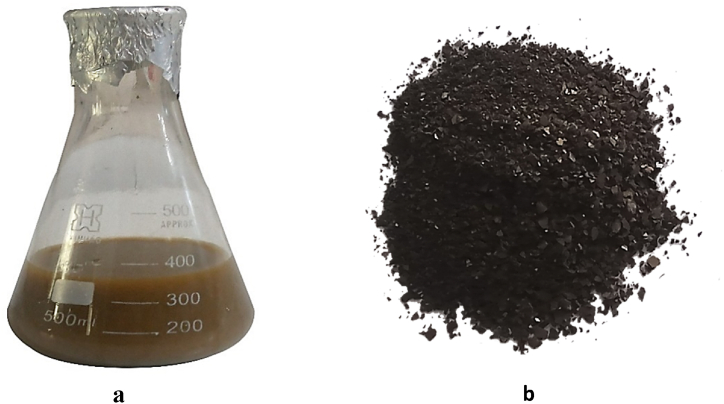
Silver nanoparticles a) color changes confirmed nanoparticle synthesis b) fabricated nanoparticles in dry powder form.

The formation of silver nanoparticles through green synthesis was visually observed, as presented in Fig. 1 a & b). The color of the solution altered after adding AgNO_3_ to the *Alnus nitida* leaves extract. The formation of a precipitate at the bottom showed the presence of silver nanoparticles. The silver nanoparticles were lipolyses to obtain the powder form of nanoparticles.

#### 5.1.2 UV–visible spectroscopy

**Fig. 2:**
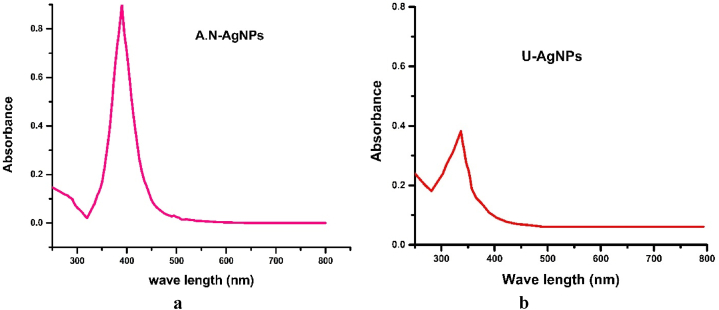
UV–Vis absorption spectra of a) silver nanoparticles (A.N-AgNPs) and b) urea-doped silver nanoparticles (U-AgNPs) using *Alnus nitida*-based plant extract.

The confirmation of silver nanoparticles formation was accomplished through the use of a UV-VIS spectrophotometer, and the corresponding spectra are presented in Fig. 2a and b). The wavelength was adjusted in the range of 250–800 nm. The silver nanoparticles showed an absorbance peak at 400 nm which is an important feature of metallic nanoparticles. The UV–vis spectra provided strong evidence that A.N-AgNPs were synthesized rapidly within a few minutes, indicating that *Alnus nitida* significantly enhances the green synthesis of silver nanoparticles. The green synthesis of AgNPs was also observed for *Megaphrynium macrostachyum* leaf extract [34]. The U-AgNPs exhibited a peak at 336 nm, demonstrating the doping effect of urea on the silver nanoparticles.

#### 5.1.3 SEM and TEM

**Fig. 3:**
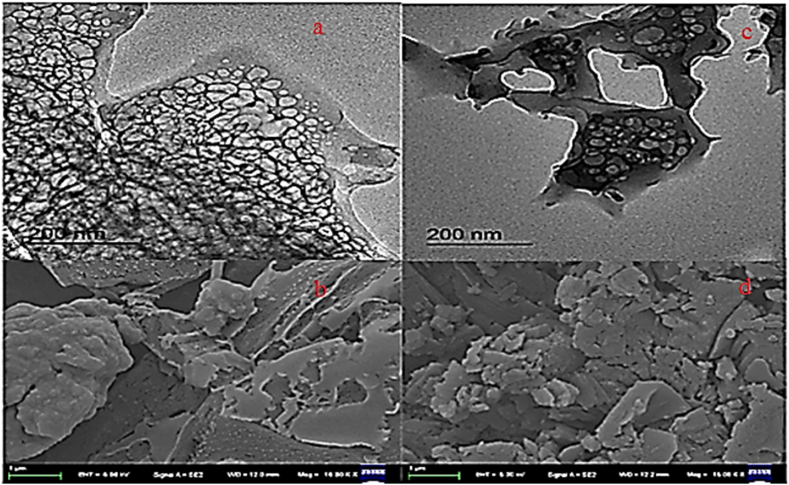
TEM (a, b) and SEM images (c, d) of A.N-AgNPs and U-AgNPs.

SEM images clearly distinguished that greenly synthesized silver nanoparticles using *Alnus nitida* extract were spherical with a diameter range of less than 100 nm. Further, an insight into the morphology and size details of A.N-AgNPs and U-AgNPs nanoparticles was provided by TEM. The TEM images also suggested the spherical shape of A.N-AgNPs. The urea-doped silver nanoparticles showed a crystalline shape, indicating that urea was successfully doped with silver nanoparticles. The image obtained in this study showed a greater resemblance with the SEM and TEM results of Rautela [35]. The TEM images are presented in Fig, 3 a&b whereas SEM images are given in Fig. 3 c&d.

#### 5.1.4 EDX spectrum

Table 1 summarizes its elemental composition of A.N-AgNPs. A pronounced signal of Ag was detected in their characteristic region due to the surface plasmon resonance, which is a typical feature of metallic silver nanoparticles, displaying a strong peak signal at 3.5 keV. Usually, the typical Ag peak is exhibited at 3 KeV [36]. The analysis showed a relative composition (in percent) of various elements as; C (41.29%), O (36.17%), N (17.49%), and Ag (5.05%). Table 2 summarizes its elemental composition urea-doped silver nanoparticles. The EDX spectra recorded at 3 keV depicted an optical absorption peak of silver in the synthesized nanoparticles. Here the depeicted percent elemental composition was; C (25.95%), O (33.93%), N (39.74%), and Ag (0.38%). The surface of silver nanoparticles was bound to other elements which acted as capping agents [37].

**Table 1:** The chemical composition of A.N-AgNPs.

Elements	Line type	Apparent concentration	k ratio	wt%	wt% Sigma	Standard sample label	Manufacturer standard
C	K line system	41.67	0.41669	41.29	0.48	C Vit	Yes
N	K line system	23.10	0.04112	17.49	0.70	BN	Yes
O	K line system	30.85	0.10381	36.17	0.47	SiO2	Yes
Ag	L line system	6.01	0.06011	5.05	0.23	Ag	Yes
Total:				100.00

**Table 2:** The chemical composition of urea-doped silver nanoparticles.

Elements	Line type	Apparent concentration	k ratio	wt%	wt% Sigma	Standard sample label	Manufacturer standard
C	K line system	41.45	0.41450	25.95	0.29	C Vit	Yes
N	K line system	144.61	0.25746	39.74	0.45	BN	Yes
O	K line system	41.29	0.13893	33.93	0.39	SiO2	Yes
Ag	L line system	0.75	0.00748	0.38	0.10	Ag	Yes
Total:				100.00			

**Fig. 4:**
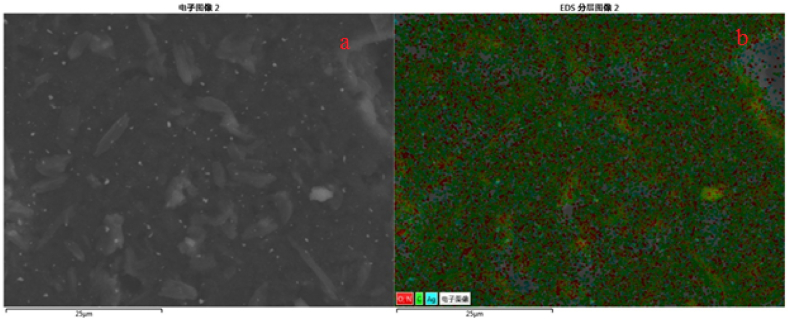
FESEM-EDX mapping of A.N-AgNPs (a & b) at different magnification.

**Fig. 5:**
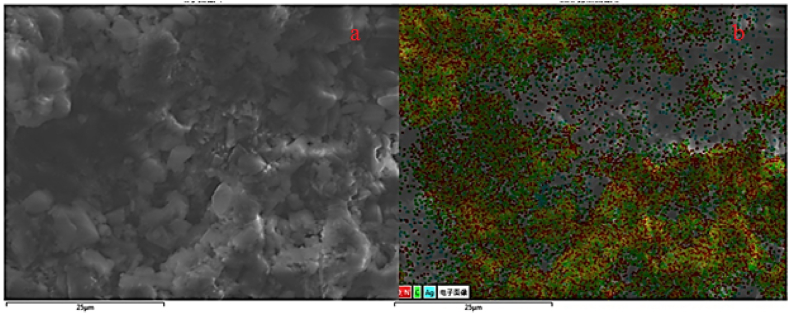
FESEM-EDX mapping of U-AgNPs (a & b) at different magnification.

Elemental mapping of A.N-AgNPs was performed by FESEM-EDX and the images are displayed in Fig. 4a and b showing the presence of 25% Ag and 75% other metals. The elemental analysis of U-AgNPs was also conducted using FESEM-EDX, which revealed the presence of 5% Ag in U-AgNPs as given in Fig. 5a and b.

#### 5.1.5 Powder X-ray diffraction (PXRD)

**Fig. 6:**
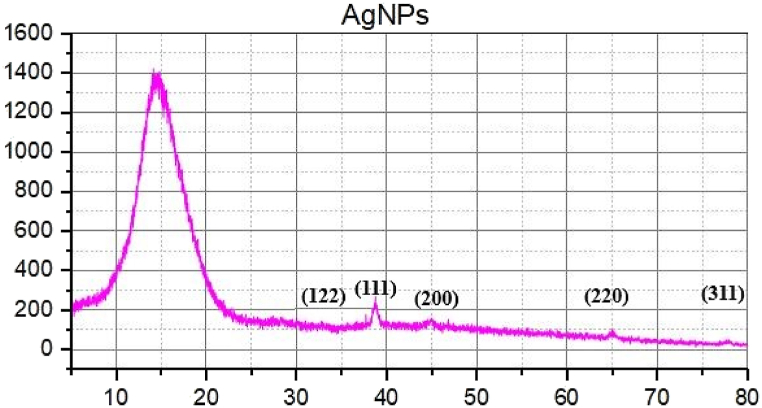
PXRD diffractogram of silver nanoparticles.

**Fig. 7:**
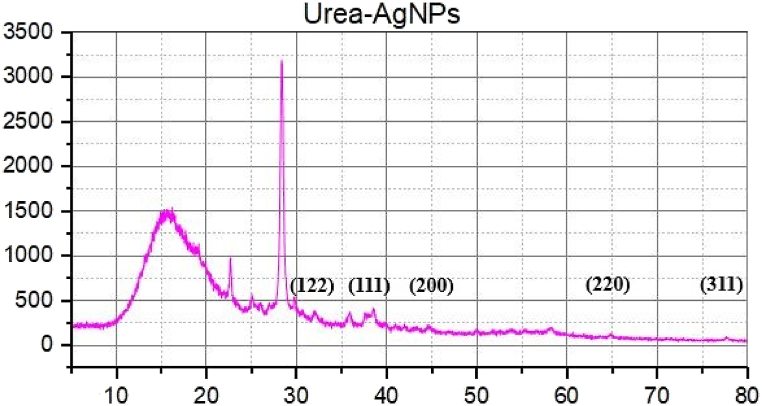
PXRD diffractogram of urea-doped silver nanoparticles.

The crystalline nature of silver nanoparticles (Fig. 6) and urea-doped A.N-AgNPs (Fig. 7) was confirmed by PXRD analysis. The diffractograms of A.N-AgNPs and U-AgNPs displayed distinct characteristic peaks, providing strong evidence for the crystalline nature of the synthesized nanoparticles. The diffraction peaks of A.N-AgNPs were observed at 32.5°, 38.3°, 44.4°, 64.6°, and 77.8° corresponding the Ag indices (122), (111), (200); (220) and (311). The U-AgNPs diffraction peaks at 32.5°, 38.3°, 44.4°, 64.6° and 77.8° corresponds to the (122), (111), (200), (220) and (311) reflection planes, respectively indicating the presence of silver. The presence of Bragg peaks was due to the leaf extract of *Alnus nitida* containing organic compounds. They are responsible to reduce the silver ions and stabilize the resultant nanoparticles. Ibrahim and Roopan et al. have reported similar PXRD results for silver nanoparticles [38,39].

## 6 Growth and morphological characteristics of wheat seed cultures

A.N-AgNPs exhibited minimal effects on seed germination at a concentration of 0.75 μg/ml. However, a slight increase in fresh weight was observed as the concentration of nanoparticles was increased to 1.5 μg/ml. The seeds demonstrated better growth at higher concentrations of 3 and 6 μg/ml compared to lower dosages. It was found that the wheat seeds gradually adapted to the nanoparticles and induced metabolite production, as compared to the control group (evident from our HPLC analysis). Previousely, the impact of nanoparticles on seed growth has been demonstrated, for instance, Shaikhaldein et al. (2020) investigated the impact of silver nanoparticles on *Maerua oblongifolia*. They have added various concentrations of AgNPs (0, 10, 20, 30, 40, or 50 mgL^−1^) to the MS medium. The shoots of *M. oblongifolia* (2–3 cm) were grown in the enriched AgNPs medium, and growth parameters such as length, weight, height, and number were measured after 6 weeks of *in vitro* shoot regeneration. The study found that treatment with 20 mgL^−1^ AgNPs significantly enhances shoot length, shoot number, dry weight, fresh weight, and chlorophyll content [40]. In our study, we observed seed inhibition in a few groups during the initial stage, particularly at the highest concentration of 15 μg/ml. After 40 days, these seeds exhibited smaller fresh weight (0.33 ± 0.39 g) and dry weight (0.11 ± 0.21 g) compared to those exposed to lower AgNPs concentrations. The use of nanoparticles in different concentrations can be either effective or harmful for plant biomass and metabolites, as increasing nanoparticle concentration is associated with toxicity in plants. The FW was reduced as the concentration was increased to 15 μg/ml, showing the toxicity of silver nanoparticles at high concentration. A similar study by Yang et al. (2018) also demonstrated the toxicity of silver nanoparticles in wheat plants, where different concentrations of AgNPs (20, 200, and 2000 mg kg^−1^) were applied in the field for wheat growth (*Triticum aestivum* L.). The results showed that increasing AgNPs dosage causes severe phytotoxicity, including shorter plant height, lower biomass, and lower grain weight [41].

In contrast, the treatment using U-AgNPs showed a more significant effect on the growth of seeds, fresh weight, dry weight, and length of roots. The results showed that the seedlings had the highest fresh weight (FW) on day 30th (2.7 ± 0.68 and 2.8 ± 0.69 g; 2.8 ± 0.45 and 3.1 ± 0.45 g) when supplemented with 3 and 6 μg/ml of A.N-AgNPs and U-AgNPs, respectively. The most significant improvement in FW was recorded due to the adaptation of wheat plants with nanoparticles. Ali et al. (2019) conducted a study on the production of biomass, antioxidants and secondary metabolites using *in vitro* callus cultures of *Caralluma tuberculata*. They used various concentrations of AgNPs and plant growth regulators (PGRs) and found that the combination of AgNPs and PGRs significantly affected the callus proliferation and considerably enhances the callus biomass in the Murashige and Skoog media. The highest dry (0.051 g/L) and fresh (0.78 g/L) callus biomass were reported *in vitro* cultures at 60 μg/L of AgNPs in combination with 0.5 mg/L 2,4-D plus 3.0 mg/L BA [42]. In our study, the lowest fresh weight on day 10th (FW = 0.24 ± 0.34 and 0.25 ± 0.31) was observed in the group that was supplemented with 0.75 μg/ml of A.N-AgNPs and U-AgNPs, compared to the control group. However, these applications still demonstrated a significant improvement in FW, with the effect of U-AgNPs being considerably higher than that of A.N-AgNPs. On day 20th, the group grown under the effect of 3 and 6 μg/ml of A.N-AgNPs and U-AgNPs, respectively, had the highest FW of 2.5 ± 0.59, 2.7 ± 0.72; and 2.7 ± 0.12, 2.9 ± 0.52 g respectively. The seedlings displayed noticeable improvement in the first few days, after the application of nanoparticles. By day 30th, the wheat plants treated with nanoparticles had exhibited a more significant increase in biomass compared to the control group.

Similarly, U-AgNPs at concentrations of 3 and 6 μg/ml induced maximum growth of roots and shoots, while at 15 μg/L, seed growth was inhibited, resulting in less biomass production. The longest roots and biomass were observed in seedlings treated with 6 μg/ml U-AgNPs, and root length was positively affected with a treatment starting at 1.5 μg/ml. However, shoot length was negatively affected as the concentration was increased to 15 μg/ml. Zia et al. (2020) investigated the effect of silver nanoparticles on the number of roots and shoots in *cv. Noblessa*, *cv. Antigua, and cv. Mariposa.* They found that supplementing the MS medium with lower concentrations of AgNPs (6 mg/L) increased the number of shoots per plant. High regeneration shoot rates were observed at 8 mg/L, while AgNPs at 12 mg/L increased the number and length of roots per plant compared with the control. Additionally, the fresh and dry weight of regenerated plants was significantly (P \2 0.05) enhanced at 6 mg/L of AgNPs [43].

The dry weight (DW) of the newly emerged seedlings was incredibly improved to 0.38 ± 0.34, 0.24 ± 0.17 g, after U-AgNPs treatment at 3 and 6 μg/ml on day 10th compared to the control group (0.18 g). The maximum DW was observed on days 20th and 30th at a concentration of 6 μg/ml (0.40 ± 0.34, 0.41 ± 0.17 g, respectively). Similarly, A.N-AgNPs showed a DW of 0.37 ± 0.35 b, 0.38 ± 0.43 g at the same concentrations. On day 40th, the maximum DW was observed after applying 6 μg/ml of U-AgNPs (0.27 ± 0.46 g) and A.N-AgNPs (0.25 ± 0.92 g). Nevertheless, increasing the concentration to 15 μg/ml on day 40th resulted in a reduction in the DW of the wheat plants **(**Table 3**)**. In another study, Vannini et al. (2014) investigated the effects of 1 and 10 mg/L AgNPs on germinating *Triticum aestivum* L. seedlings and found that the application of 10 mg L−1 AgNPs had an adverse effect on seedling growth and morphologically modified the root tip of cells [44]. Our findings demonstrated that the wheat seedlings treated with A.N-AgNPs and U-AgNPs at concentrations of 3 and 6 μg/ml, respectively, have the highest fresh and dry weights.

**Table 3:** Effect of silver and urea dopped silver nanoparticles on physiological parameters of wheat seedlings.

Doses (μg/ml)			FW (g) at day					DW (g) at day				Color at day			
			0	10	20	30	40	10	20	30	40	10	20	30	40
Control			0.9	0.22 ± 0.12^a^	0.24 ± 0.19^a^	0.33 ± 0.32^b^	0.35 ± 0.37^b^	0.13 ± 0.09^a^	0.15 ± 0.06^a^	0.18 ± 0.12^a^	0.13 ± 0.05^a^	G	Y/G	Y	Y/G
A.N-AgNPs	**0.75**		0.9	0.24 ± 0.34^b^	2.2 ± 0.37^b^	2.3 ± 0.36^b^	0.34 ± 0.25^c^	0.17 ± 0.11^a^	0.35 ± 0.30^b^	0.15 ± 0.14^a^	0.14 ± 0.06^a^	G	Y/G	Y	Y/G
	**1.5**		0.9	0.27 ± 0.37^b^	2.4 ± 0.41^d^	2.5 ± 0.52^d^	0.34 ± 0.27^c^	0.25 ± 0.14^a^	0.37 ± 0.34^b^	0.17 ± 0.21^a^	0.16 ± 0.19^a^	G	Y/G	Y	Y/G
	**3**		0.9	0.27 ± 0.21^c^	2.5 ± 0.59^d^	2.7 ± 0.68^e^	0.36 ± 0.29^b^	0.27 ± 0.19^a^	0.36 ± 0.42^d^	0.16 ± 0.13^a^	0.18 ± 0.22^c^	G	G	G	G
	**6**		0.9	0.28 ± 0.41^d^	2.7 ± 0.72^e^	2.8 ± 0.69^e^	0.39 ± 0.34^b^	0.28 ± 0.35^b^	0.37 ± 0.35^b^	0.38 ± 0.43^d^	0.25 ± 0.92^d^	G	G	G	G
	**15**		0.9	0.21 ± 0.27^c^	2.0 ± 0.6^d^	2.1 ± 0.70^e^	0.33 ± 0.39^b^	0.14 ± 0.08^a^	0.16 ± 0.04a	0.12 ± 0.06^a^	0.11 ± 0.21^c^	G	Y/G	Y/G	Y
U-AgNPs	**0.75**		0.9	0.25 ± 0.31^b^	2.3 ± 0.4^d^	2.3 ± 0.45^d^	0.34 ± 0.24^c^	0.17 ± 0.04^a^	0.35 ± 0.45^c^	0.15 ± 0.03^a^	0.14 ± 0.05^a^	G	G	Y/G	Y/G
	**1.5**		0.9	0.28 ± 0.32^b^	2.4 ± 0.3^b^	2.5 ± 0.37^b^	0.36 ± 0.26^c^	0.19 ± 0.18^a^	0.37 ± 0.42^d^	0.19 ± 0.21^c^	0.13 ± 0.23^c^	G	G	Y/G	Y
	**3**		0.9	0.30 ± 0.37^b^	2.7 ± 0.12^a^	2.8 ± 0.45^d^	0.36 ± 0.67^e^	0.38 ± 0.34^b^	0.18 ± 0.46^d^	0.37 ± 0.27^c^	0.15 ± 0.35^b^	G	G	G	G
	**6**		0.9	0.31 ± 0.28^c^	2.9 ± 0.52^d^	3.1 ± 0.45^d^	0.43 ± 0.56^d^	0.24 ± 0.17^a^	0.40 ± 0.34^b^	0.41 ± 0.17^a^	0.27 ± 0.46^d^	G	G	G	G
	**15**		0.9	0.27 ± 0.26^a^	2.1 ± 0.62^e^	2.2 ± 0.45^d^	0.32 ± 0.46^d^	0.15 ± 0.45^d^	0.31 ± 0.27^a^	0.11 ± 0.02^a^	0.12 ± 0.53^d^	Y/G	Y/G	Y/G	Y

Fresh weight, DW dry weight, Y yellow, G green.

The superscripted letters (a, b, c, d etc.) shows signifcant diference between variables FW.

Values with diferent letters represent signifcant diference (P \2 0.05).

### 6.1 Effect of A.N-AgNPs and U-AgNPs on physiological parameters of newly germinated wheat plants

Table 3, summarizes the effects of the fabricated silver nanoparticles and urea-doped silver nanoparticles with Thidiazuron supplementation on various physiological parameters on 10, 20,30, and 40 days old plant produced from the wheat seeds in Murashige and Skoog medium.

### 6.2 Phytochemical analysis of wheat seeds growth under the stress of A.N-AgNPs and U-AgNPs

#### 6.2.1 Total phenolic content

The total phenolic content (TPC) of wheat plant samples was evaluated on days 10, 20, 30, and 40, and the results are shown in Table 4. TPC was found to be lower on day 10th with the application of 1.5 μg/ml A.N-AgNPs and U-AgNPs. The lowest TPC was recorded after supplementation with 0.75 μg/ml A.N-AgNPs and U-AgNPs. Overall, TPC on days 10, 20, 30, and 40th was higher in plants supplemented with 3 μg/ml A.N-AgNPs and U-AgNPs (251.32 ± 4.97, 260.32 ± 5.16, 265.88 ± 3.62, 268.34 ± 11.44; 261.11 ± 4.57, 265.66 ± 6.17, 273.72 ± 3.62, 271.44 ± 11.63 μg GAE/g of DW) than in the control group (TPC = 211.44 ± 2.82, 223.17 ± 3.75, 231.57 ± 3.62, 240.41 ± 4.16 μg GAE/g of DW). However, the highest TPC was produced in 30, and 40 days old wheat plants after supplementation with 6 μg/ml U-AgNPs and A.N-AgNPs (DW = 289.43 ± 3.62, 295.95 ± 12.62; 274.44 ± 3.62, 277.54 ± 11.92 μg GAE/g of DW respectively. Likewise, at days 20, 30, and 40th, the addition of varying concentrations of A.N-AgNPs and U-AgNPs, ranging from 0.75 μg/ml (low) to 15 μg/ml (lowest), 1.5 μg/ml (slightly higher), 3 μg/ml (higher), and 6 μg/ml (highest) μg/ml, resulted in a notable improvement in the TPC. Among these days, the TPC was highest on day 40 after 6 μg/ml of U-AgNPs were added to the media. The findings unequivocally demonstrate that the addition of U-AgNPs to the media at a concentration of 6 μg/ml has yielded a remarkable enhancement in the phenolic content. This concentration can be deemed as the optimal level for the purposes of this study. The effects of silver nanoparticles (AgNPs) were assessed on the growth of *pearl millet* (*P. glaucum* L.), an economically significant crop. *In vitro* experiments were conducted using MS basal medium with different concentrations of AgNPs (T1 = control, T2 = 20 ppm, T3 = 40 ppm, T4 = 60 ppm, and T5 = 80 ppm). The results of this study demonstrate that the seedlings treated with T3 exhibited significantly higher total phenolic content (0.56 ± 0.0152 μg/mg) compared to the control and T2. As the doses of AgNPs were increased from T3 to T5, there were significant impacts (p ≤ 0.01) on the accumulation of phenolic compounds. These effects caused a notable decrease in total phenolic content in T4 and T5, which amounted to 12.1% and 35.2% respectively, compared to T1. These findings strongly suggest that higher doses of AgNPs induced more stress on the seedlings, resulting in the accumulation of fewer phenolic compounds. It was evident that high doses of AgNPs acted as stressors, hindering the accrual of TPC [45]. The high concentration of U-AgNPs and A.N-AgNPs was inadequate for the wheat plant as excess nanomaterial failed to cope with cells in the media. A study was carried out to investigate the toxic effect of increasing concentrations of AgNPs on wheat plants. The phenolic content in the plants increased when subjected to stripe rust stress (5.6 μg·mg−1 F W); however, the application of AgNPs led to a decline in the phenolic content. The maximum reduction (3.7 μg mg^−1^ FW) was observed in plants treated with 75 ppm of AgNPs. These findings suggest that the presence of AgNPs may negatively affect the phenolic content in wheat plants, particularly at higher concentrations [46]. On the other hand, lower concentrations of AgNPs have been found to strongly promote the production of secondary metabolites in the growth media of wheat seeds. However, high concentrations of 25 nm AgNPs had a toxic effect when supplemented with the plant *Oryza sativa*, as they were found to break the cell wall and damage the vacuoles of root cells [47]. Mirzajani et al. (2013) reported that root cell penetration did not occur in *O. sativa* in the presence of low concentrations of AgNPs (30 μg/ml). The lowest TPC content was recorded after increasing AgNPs to 15 μg/ml. Thus, higher concentrations were found to have a toxic effect, destroying the cell structure. Previously, a study showed that a concentration of 30 μg/ml enhanced root growth, while a concentration of 60 μg/ml suppressed cell growth ability. These findings indicate that the effects of AgNPs on plant growth can be highly dependent on the concentration [48]. AgNPs-elicited hairy roots and a higher TPC concentration were produced in the cultures of *Cucumis anguria* [49]*.*

**Table 4:** Total phenolic content in wheat plants and control group under the treatment of different concentrations of A.N-AgNPs and U-AgNPs-based nano fertilizers.

*Alnus nitida*-based nanoparticles	Samples (μg/mL)	Total phenolic content **(**μg GAE/g of DW**)**			
		Day 10	Day 20	Day 30	Day 40
	Control	211.44 ± 2.82	223.17 ± 3.75	231.57 ± 3.62	240.41 ± 4.16
A.N-AgNPs	0.75	220.67 ± 3.14	223.43 ± 2.37	225.33 ± 12.67	229.77 ± 5.35
	1.5	237.98 ± 3.62	238.44 ± 4.15	240.22 ± 3.62	242.61 ± 6.62
	3	251.32 ± 4.97	260.32 ± 5.16	265.88 ± 3.62	268.34 ± 11.44
	6	269.74 ± 3.22	271.66 ± 4.74	274.44 ± 3.62	277.54 ± 11.92
	15	243.47 ± 4.16	239.66 ± 3.64	230.35 ± 3.62	220.85 ± 2.35
U-AgNPs	0.75	225.13 ± 2.47	230.90 ± 2.60	232.45 ± 7.85	239.97 ± 3.19
	1.5	245.45 ± 5.47	250.76 ± 4.29	255.35 ± 3.62	263.44 ± 4.28
	3	261.11 ± 4.57	265.66 ± 6.17	273.72 ± 3.62	271.44 ± 11.63
	6	279.74 ± 5.39	280.41 ± 5.46	289.43 ± 3.62	295.95 ± 12.62
	15	249.34 ± 4.71	244.66 ± 4.17	240.12 ± 3.62	241.34 ± 3.47

DW = Dry weight, GAE = Gallic acid equivalents.

Values are means ± SD of three determinations. Means within each column with different letters differ significantly (*P* \2 0.05).

#### 6.2.2 Total flavonoids content

In this study, the total flavonoid content (TFC) in wheat plant samples was evaluated on different days (10, 20, 30, and 40) following various concentrations supplementation with A.N-AgNPs and U-AgNPs, and the results are presented in Table 5. At day 10, the application of 1.5 μg/ml A.N-AgNPs and U-AgNPs steered to a lower TFC production. Generally, TFC was higher (80.33 ± 5.15, 86.22 ± 5.35, 90.11 ± 6.67, 93.22 ± 91.14±6.65, 94.55 ± 6.75, 97.22 ± 5.93, 102.43 ± 6.63 μg GAE/g of DW) on day 10, 20, 30, and 40 after being supplemented with 3 μg/ml A.N-AgNPs and U-AgNPs, respectively, compared to the control group (TFC = 62.12 ± 3.35, 66.67 ± 3.75, 68.47 ± 4.14, 71.33 ± 4.32 μg GAE/g of DW). The highest TFC (127.34 ± 7.86 μg GAE/g of DW) was reported in the 40-day-old wheat plant after supplementation with 6 μg/ml U-AgNPs. In the previous study, biogenic silver nanoparticles were synthesized using *Aloe vera* leaf extracts and their effects on the growth characteristics and flavonoid contents of wheat were studied by exposing the plants to different concentrations of Ag nanoparticles and Ag (0, 40, and 80 ppm). Results showed that the application of 40 and 80 ppm of both Ag nanoparticles and Ag significantly increased the flavonoid contents at 300 and 330 nm wavelengths, while no effect was observed on flavonoid contents at the 270 nm wavelength. These findings suggest that the use of Ag nanoparticles can enhance the flavonoid contents of wheat, which could have potential benefits for human health [50]. Similarly, on days 20 and 40, TFC was enhanced following supplementation with different concentrations of A.N-AgNPs and U-AgNPs, such as 0.75 (lowest), 1.5 (slightly high), 3 (high), 6 (highest), and 15 (lowest) μg/ml. Among these days, TFC was highest on day 40 after supplementation with 6 μg/ml U-AgNPs following by AgNPs. The lowest TFC content (78.44 ± 4.75, 71.34 ± 4.32, 69.33 ± 3.57, 66.12 ± 4.75; 82.45 ± 4.68, 79.34 ± 4.13, 79.55 ± 4.42, 75.22 ± 4.55 μg GAE/g of DW was recorded after enhancing the concentration of AgNPs, U-AgNPs to 15 μg/ml compared to other dosages. In a study, different AgNPs concentrations such as 25 ppm and 50 ppm showed remarkably positive impact on the production of chlorophyll content, soluble sugars, and proteins compared to both the control group and AgNO_3_. However, the study also revealed that a higher concentration of 100 ppm of AgNPs led to a relatively lower level of total phenolic content, flavonoids, compounds, total reducing potential, total antioxidant capacity, and DPPH. The use of high concentrations of AgNPs may result in negative impacts on other parameters [51]. In our study, we reported that high concentration of U-AgNPs and A.N-AgNPs was inadequate for the wheat plant as excess nanomaterial failed to cope with cells in the media. Previously a study reported the toxicity of AgNPs to the plants that enhanced ROS production and altered the plants' anatomical and genetic performances [52]. The excess production of ROS due to AgNPs exposure is causing several toxic effects on plants, including; peroxidation of polyunsaturated fatty acids (referred to as lipid peroxidation), damage to the permeability of cell membranes, and modification of the structure of the cells. Furthermore, it directly damages DNA and protein and causes growth inhibition and potential cell death in plants [53,54]. Typically, plant secondary metabolites accumulate during the late growth phases. However, by providing elicitors to the media, biosynthesis can be stimulated during the initial stages as a counter-defense mechanism. The study reported that the enhancing effects of AgNPs were observed at every growth stage. Providing AgNPs on day 10 resulted in the highest total flavonoid content, with a value of 11.85 mg QUE/g DW [55].

**Table 5:** Total flavonoid content in wheat plants and control group under the treatment of different concentrations of A.N-AgNPs and U-AgNPs-based nano fertilizers.

*Alnus nitida*-based nanoparticles	Samples (μg/mL)	Total flavonoids content **(**μg GAE/g of DW**)**			
		Day 10	Day 20	Day 30	Day 40
	Control	62.12 ± 3.35	66.67 ± 3.75	68.47 ± 4.14	71.33 ± 4.32
A.N-AgNPs	0.75	73.27 ± 3.97	74.77 ± 4.13	74.88 ± 4.85	75.11 ± 4.76
	1.5	76.36 ± 4.48	78.24 ± 4.85	81.44 ± 5.45	84.22 ± 5.67
	3	80.33 ± 5.15	86.22 ± 5.35	90.11 ± 6.67	93.22 ± 6.44
	6	103.23 ± 6.67	104.23 ± 6.85	107.57 ± 7.46	111.47 ± 7.34
	15	78.44 ± 4.75	71.34 ± 4.32	69.33 ± 3.57	66.12 ± 4.75
U-AgNPs	0.75	75.22 ± 4.98	75.91 ± 4.67	76.20 ± 4.85	77.19 ± 5.56
	1.5	81.33 ± 5.54	82.13 ± 5.86	86.45 ± 5.45	89.40 ± 5.83
	3	91.14 ± 6.65	94.55 ± 6.75	97.22 ± 5.93	102.43 ± 6.63
	6	117.44 ± 7.47	121.89 ± 7.56	124.57 ± 6.46	127.34 ± 7.86
	15	82.45 ± 4.68	79.34 ± 4.13	79.55 ± 4.42	75.22 ± 4.55

DW = Dry weight, GAE = Gallic acid equivalents.

Values are means ± SD of three determinations. Means within each column with different letters differ significantly (P \2 0.05).

#### 6.2.3 Chlorophyll content analysis

**Fig. 8:**
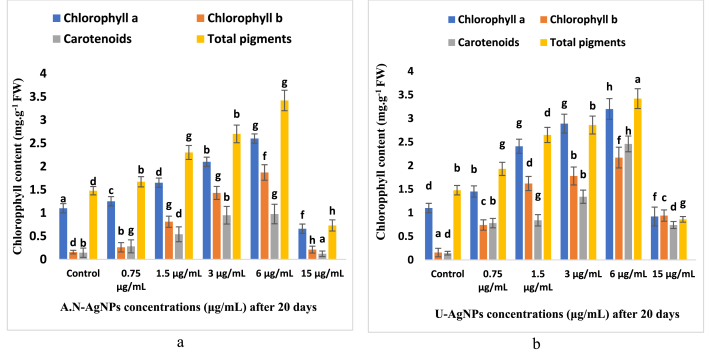
The effects of A.N-AgNPs (a) and U-AgNPs (b) on chlorophyll content after 20 days of wheat treatment under *in vitro* conditions. Superscripted letters, such as a, b, c, d, etc., are used to indicate significant differences between variables. Values of different letters showed a statistically significant difference between (P \2 0.05).

The silver nanoparticles and urea-doped silver nanoparticles showed a significant aspect in the recent pigments study in wheat leaves. The chlorophyll *a*, chlorophyll *b*, carotenoids, and total pigments in the leaf extract of wheat at 3 μg/ml were 2.1 ± 0.62, 1.43 ± 0.56, 0.95 ± 0.85, and 2.7 ± 0.73 mg.g-1 FW, respectively. The total chlorophyll content after wheat treatment at 6 μg/ml was remarkably improved such as 2.6 ± 0.63, 1.87 ± 0.33, 0.976, ±0.15 and 2.91 ± 0.86 mg.g-1 FW in the photosynthetic pigment compared to the control and A.N-AgNPs. Therefore, this treatment could be ideal for enhancing chlorophyll content. Previously, *Stevia (Stevia rebaudiana B.)* was exposed to silver nanoparticles at varying concentrations, including 0 mg/L, 12.5 mg/L, 25 mg/L, 50 mg/L, 100 mg/L, and 200 mg/L. The study revealed significant differences in the chlorophyll *a*, *b*, and total contents among the different concentrations of AgNPs. Generally, chlorophyll content was increased starting from 25 mg/L. The control treatment and the less concentration of AgNPs (12.5 mg/L) resulted in lower levels of chlorophyll *a*, *b*, and total contents [56]. The silver nanoparticles were biosynthesized using *Ochradenus arabicus* and their physiological effects were checked on *Maerua oblongifolia* raised *in vitro*, Murashige and Skoog medium was supplemented with varying concentrations of AgNPs, including 0 mg/L, 10 mg/L, 20 mg/L, 30 mg/L, 40 mg/L, and 50 mg/L, for 6 weeks. The study results revealed that the plants treated with 20 mg/L AgNPs exhibited an increase in shoot length, shoot number, dry weight, fresh weight, and chlorophyll content. Based on these findings, it can be suggested that AgNPs have the potential to serve as a growth-promoting agent for *in vitro* raised *Maerua oblongifolia* [40]. The treatment U-AgNPs at 15.6 μg/ml highly reduced the pigment content to 0.66 ± 0.19, 0.21 ± 0.06, 0.12 ± 0.04, 0.73 ± 0.25 mg.g-1 FW. The results showed that enhancing silver nanoparticle concentration negatively affects the formation of photosynthetic pigments. A study was conducted to explore the effects of AgNPs or AgNO_3_ supplementation on *in vitro* potato plant cultures. The findings demonstrated that the total chlorophyll content increased significantly in cultures treated with 2 mg of both AgNO_3_ and AgNPs compared to the control group. However, total chlorophyll content was reduced as the concentration of the supplementation increased to 10 and 20 mg. Notably, the AgNPs treatment group exhibited a more distinct reduction in total chlorophyll content compared to the AgNO_3_ treatment group [57]. The chlorophyll *a*, chlorophyll *b*, carotenoids, and total pigments were tremendously increased to 3.2 ± 0.92, 2.17 ± 0.76, 2.46 ± 0.83, and 3.42 ± 0.98 mg.g-1 FW after treatment wheat seeds (U-AgNPs) at 6 μg/ml. At the same time, the urea-doped silver nanoparticles showed fewer adverse effects with less improvement of the chlorophyll contents. The study presented here provided evidence that both A.N-AgNPs and U-AgNPs significantly improved pigment content. Notably, U-AgNPs were found to be highly effective under *in vitro* conditions, as shown in Fig. 8 a & b. This may be due to the lower silver nanoparticle content in urea-doped silver nanoparticles. Verma et al. have biologically synthesized the silver nanoparticles. The seed treatment method used different concentrations of silver nanoparticles, such as 0, 15, 30, 60, 120, 240, and 480 mg/L. The low concertation of silver nanoparticles enhanced the chlorophyll content, while higher concentrations showed toxicity and reduced the total chlorophyll [58].

#### 6.2.4 DPPH free radical scavenging activity

**Fig. 9:**
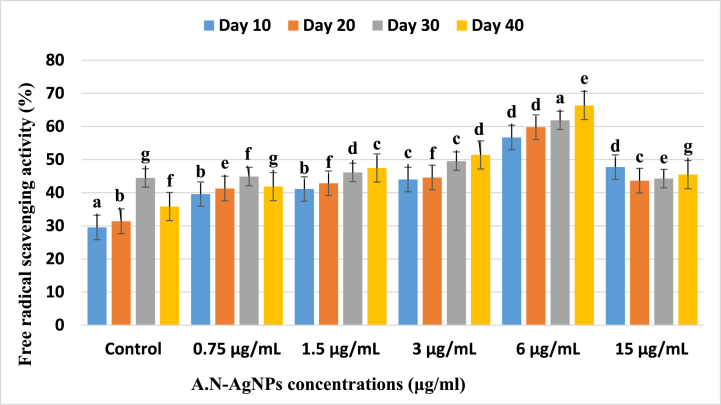
The effect of different concentrations of silver nanoparticles (A.N-AgNPs) on 10-, 20-, 30-, and 40-day-old wheat plant seed grown in media. The highest % free radical scavenging activity (67.36%) was reported on day 40 at 6 μg/ml A.N-AgNPs compared to the control and diverse group. Superscripted letters, such as a, b, c, d, etc., are used to indicate significant differences between variables. Values of different letters showed a statistically significant difference between (P \2 0.05).

The antioxidant activity was performed using the protocol of % DPPH scavenging capacity the results were depicted in Fig. 9. The wheat plant samples treated with A.N-AgNPs were extracted after 10, 20, 30, and 40 days, and the antioxidant activity was measured. At a concentration of 3 μg/ml of A.N-AgNPs, the antioxidant activity on day 10 was 43.98%, whereas the maximum antioxidant activity was observed on day 20 at a concentration of 6 μg/ml. A study conducted on *Echium amoenum* plants treated with various concentrations (25 and 50 ppm) of silver nanoparticles revealed a significant improvement in DPPH free radical scavenging in the treatment groups as compared to the control group, thereby demonstrating the efficacy of AgNPs [59]. The lowest activity (47.75, 43.63, 44.26, 45.46%) was recorded in 10-, 20-, 30-, and 40-days-old wheat plants derived from media with 15 μg/ml of A.N-AgNPs. In a study, silver nanoparticles were synthesized using pod extract of *Cola nitida* at diverse concentrations (25, 50, 75, 100, and 150 ppm) and tested their DPPH free radical scavenging activity. The results confirmed that various concentrations of AgNPs significantly improved the antioxidant activity of *A. caudatus*, except for the treatment with 150 ppm AgNPs, which demonstrated lower antioxidant activity than the control group grown with water [60]. These findings support our results, which showed a reduction in activity when the concentration of silver nanoparticles was increased *in vitro*. In addition, the control group demonstrated the lowest levels of antioxidant activity at 29.51%, 31.39%, 44.46%, and 35.83%. However, supplementing with silver nanoparticles (A.N-AgNPs) revealed the potential for A.N-AgNPs to enhance the antioxidant capacity of wheat seeds. The maximum antioxidant activity levels were observed in 20-, 30-, and 40-day-old wheat plants exposed to 3 μg/ml of A.N-AgNPs, reaching values of 43.98%, 44.58%, 49.54%, and 51.42%. Furthermore, increasing the concentration of A.N-AgNPs in the media up to 6 μg/ml significantly induced antioxidant potential in wheat plants, resulting in levels of 65.67%, 59.76%, 61.85%, and 66.35%. Another study showed that the antioxidant, chloroform fraction was highly effective during the determination of DPPH, ABTS, and FRAP assays, which displayed an IC_50_ value of 64.99, 69.15, and 268.52 μg/ml. Ethyl acetate extract has also shown potency in tested free radicals. The potency of both types of extracts against lipoxygenase was confirmed with IC_50_ values of 75.99 and 106.11 μg/ml, respectively. The results of the biological studies conducted on *Ilex dipyrena* showed that it is a good inhibitor of free radicals and lipoxygenase. Thus, it is very important to further investigate the plant for the isolation of significant medicinal compounds [[61], [62], [63]]. The biological activities of inorganic nanoparticles, including silver nanoparticles, can be influenced by various factors such as size distribution, shape, surface charge, coating, composition, aggregation, solubility, and capping agent. These factors have the potential to significantly impact the cellular uptake, biodistribution, stability, toxicity, and potential applications of the nanoparticles [64,65].

## 7 HPLC analyses of A.N-AgNPs and U-AgNPs -based nanoparticles

The HPLC analysis of our samples (presented in Table 6) revealed that the addition of A.N-AgNPs and U-AgNPs to the growth medium had a significant effect on the production of phenolic compounds during wheat plant growth. The plant extract obtained from *Alnus nitida* was found to contain five different antioxidant phytochemicals. As this plant is known for its greater diversity compared to wheat, it is expected that the potential compounds between the two will also differ from each other. The application of silver nanoparticles (A.N-AgNPs) and urea-doped silver nanoparticles (U-AgNPs) led to significant modifications in the quality and quantity of secondary metabolites. In comparison to the nanoparticle treatments, the control group exhibited only a single compound. The *in vitro* wheat seeds treatment at 3 and 6 μg/ml produced four significant metabolites namely Dihydroxybenzoic acids (0.6 μg/ml), Apigenin glucosidase (0.03 μg/ml), *p*-Coumaric acid (1.3 μg/ml), Sinapic acid (0.21 μg/ml); Apigenin glucosidase (0.135 μg/ml), *p*-Coumaric acid (0.138 μg/ml), Sinapic acid (0.2 μg/ml), and Ferulic acid (0.27 μg/ml) respectively. The results indicated that increasing the concentration of nanoparticles was positively correlated with the production of secondary metabolites. For instance, the effect of U-AgNPs at 3 and 6 μg/ml produced a diverse range of metabolites such as Dihydroxybenzoic acids (1.02 μg/ml), Vanillic acid (0.25 μg/ml), Apigenin glucosidase (0.245 μg/ml), Sinapic acid (0.28 μg/ml), Ferulic acid (0.2 μg/ml); Dihydroxybenzoic acids (0.66 μg/ml), Vanillic acid (0.28 μg/ml), Apigenin glucosidase (0.084 μg/ml), *p*-Coumaric acid (0.35 μg/ml), Sinapic acid (0.845 μg/ml), and Ferulic acid (0.135 μg/ml) respectively (Table 6). We also investigated that at 6 μg/ml of both A.N-AgNPs and U-AgNPs treatment resulting in six different peaks that highly induced metabolites. In comparison, the U-AgNPs treatment strongly induced the metabolic contents as compared to A.N-AgNPs. Therefore, we concluded that urea-doped silver nanoparticles might be the significant fertilizers for the wheat seed that would enhance the phenolic contents. Amjad et al. (2022) have performed HPLC that investigated five possible compounds in Crd-Id such as catechin hydrate, ellagic acid, morin, epigallocatechin gallate, and rutin. In contrast, seven possible compounds were identified in Et-Id such as malic acid, morin, epigallocatechin gallate, ellagic acid, hydrate, pyrogallol, and catechin rutin. Five possible phenolic compounds in Chl-Id were catechin hydrate, ellagic acid, epigallocatechin gallate, rutin, and morin. Previously, a study of HPLC has performed on *A. nitida* leaves which exhibited six possible phytochemicals in the Met. Ext such as chlorogenic acid, malic acid, quercetin, epigallocatechin gallate, pyrogallol, and ellagic acid. Stem bark in the Met. Ext showed phenolic compounds such as pyrogallol, ellagic acid, and epigallocatechin gallate. Six phenolic compounds were identified in the Met. Ext of seed such as vitamin C, malic acid, quercetin, epigallocatechin gallate, pyrogallol, and ellagic acid. However, five phenolic compounds were detected in the Met. Ext of root such as ellagic acid, epigallocatechin gallate, malic acid, and quercetin [66,67].

**Table 6:** High-performance liquid chromatography-based quantification of phenolic compounds induced as a result of A.N-AgNPs and U-AgNPs in the wheat seed cultures.

Sample extracts	Retention Time	No. of peaks	Wavelength	Possible compound Identity	Concentration (μg/ml)	Reference
*Alnus nitida*	2.279	1	320	Phloroglucinol	2.55	Standard
	2.677	2	320	Dihydroxybenzoic acids	0.3	Standard
	32.797	3	320	Quercetin	0.18	Standard
	35.291	4	320	Rutin	0.175	Standard
	36.758	5	320	Morin	0.2	Standard
Control	2.602	1	320	Dihydroxybenzoic acids	0.64	Standard
A.N-AgNPs (3 μg/ml)	2.2455	1	320	Dihydroxybenzoic acids	0.6	Standard
	14.493	2	320	Apigenin glucosidase	0.03	Standard
	15.283	3	320	*p*-Coumaric acid	1.3	Standard
	16.183	4	320	Sinapic acid	0.21	Standard
A.N-AgNPs (6 μg/ml)	14.350	1	320	Apigenin glucosidase	0.135	Standard
	15.084	2	320	*p*-Coumaric acid	0.138	Standard
	16.936	3	320	Sinapic acid	0.2	Standard
	17.699	4	320	Ferulic acid	0.27	Standard
U-AgNPs (3 μg/ml)	2.434	1	320	Dihydroxybenzoic acids	1.02	Standard
	13.662	2	320	Vanillic acid	0.25	Standard
	14.562		320	Apigenin glucosidase	0.245	Standard
	16.156	3	320	Sinapic acid	0.28	Standard
	17.038	4	320	Ferulic acid	0.2	Standard
U-AgNPs (6 μg/ml)	2.629	1	320	Dihydroxybenzoic acids	0.66	Standard
	13.956	2	320	Vanillic acid	0.28	Standard
	14.319	3	320	Apigenin glucosidase	0.084	Standard
	15.168	4	320	*p*-Coumaric acid	0.35	Standard
	16.971	5	320	Sinapic acid	0.845	Standard
	17.508	6	320	Ferulic acid	0.135	Standard

## 8 Conclusions and future perspectives

Medicinal plants are highly valuable in both the medical and agricultural sectors, and this study underscores their importance of utilizing leaf extracts from *Alnus nitida* to synthesize silver nanoparticles. The resulting nanoparticles were shown to be spherical, crystalline, and stable, with particle sizes reaching up to 100 nm as evidenced by UV–visible spectroscopy, PXRD, SEM, TEM, and EDX analyses. Moreover, the study highlights the potential of U-AgNPs as a significant elicitor of plant cell metabolism, prompting the production of secondary metabolites. U-AgNPs-based wheat seed cultures demonstrated a marked increase in phenolic, chlorophyll, flavonoid, and antioxidant activity levels when compared to A.N-AgNPs. The application of urea-doped silver nanoparticles could be an effective approach that produced novel secondary metabolites in plant cells. Silver nanoparticles, especially U-AgNPs, have vast potential to boost the yield of total phenolic, chlorophyll, and flavonoid contents in wheat plants. Furthermore, they effectively enhanced antioxidant activity and biomass production in seed cultures of wheat under *in vitro* conditions, signifying their potential as a tool for enhancing the growth and yield of critical crops. Silver nanoparticles hold great promise in advancing the agricultural sector and promoting sustainable crop growth. However, to fully understand the effects of these nanoparticles on wheat seedlings and their potential impact on the environment, more research is required. Future studies should focus on investigating the optimal concentration, size, and surface coating of these nanoparticles to maximize the benefits for crop growth and development. By continuing to explore the potential of silver nanoparticles and urea-doped silver nanoparticles, we can find innovative ways to improve the efficiency and sustainability of agriculture, benefiting both the environment and society.

## Author contribution statement

Muhamad Zahoor and Sajad Khan; Conceived and designed the experiments: Performed the experiments; Wrote the paper.

Rahm Sher Khan and Muhammad Zahoor, Noor Ul Islam, Tariq Khan, Rahm Sher Khan, and Sikandar Khan: Analyzed and interpreted the data.

Zar Muhammad; Riaz Ullah; Ahmed Bari: Contributed reagents, materials, analysis tools or data.

## Funding statement

The authors extend their appreciation to the researchers supporting Project number (RSP2023R346). King Saud University, Riyadh, Saudi Arabia, for financial support.

## Data availability statement

No data was used for the research described in the article.

## Declaration of interest's statement

The authors declare no conflict of interest.

## Additional information

No additional information is available for this paper.

## Declaration of competing interest

The authors declared that they do not have any conflict in publishing this research article.

## Abbreviation

A.N-AgNPs: *Alnus nitida* based silver nanoparticles
U-AgNPs: urea-doped silver nanoparticle
FTIR: Fourier transform infrared
SEM: Scanning electron microscope
TEM: Transmission electron microscopy
XRD: X-Ray Diffraction
EDX: Energy dispersive X-Ray
HPLC: High-Performance Liquid Chromatography
TDZ: Thidiazuron
MS media: Murashige and Skoog medium
DW: Dry weight
FW: Fresh weight
GAE: Gallic acid equivalents
